# Phosphorus retention, erosion protection and farmers’ perceptions of riparian buffer zones with grass and natural vegetation: Case studies from South-Eastern Norway

**DOI:** 10.1007/s13280-020-01361-5

**Published:** 2020-09-15

**Authors:** Anne-Grete Buseth Blankenberg, Eva Skarbøvik

**Affiliations:** NIBIO – the Norwegian Institute of Bioeconomy Research, Oluf Thesens vei 43, 1433 Ås, Norway

**Keywords:** Bioeconomy, Buffer Zones, Erosion, Farmers’ perceptions, Nutrient retention

## Abstract

Phosphorus retention and bank erosion was investigated in two types of buffer zones in cereal fields in Norway: zones used for grass production and zones with natural vegetation. Farmers’ views on the two types of buffer zones were collected through questionnaires and in-depth interviews. Our results indicate that the grassed buffer zones had higher levels of plant-available phosphorus and lower infiltration rates than the natural ones. Bank erosion was higher in zones with grass production than those with trees. Interviews with farmers revealed diverging opinions on the zones. Most farmers were sceptical to natural vegetation with trees, whereas farmers who had already planted trees in the riparian zones were generally satisfied. Buffer zones can have many different functions, and we conclude that a holistic approach is needed when assessing the usefulness of this measure, taking into account water quality, biodiversity and the production of food, fodder and biomass.

## Introduction

Buffer zones (BZs) are much-used mitigation measures in agricultural lands, and are most commonly designed to retain inputs of nutrients and particles from adjacent fields (e.g. Roberts et al. 2012; Stutter et al. 2019). Vegetation in these zones can also serve other functions, such as protection against bank erosion, production of biomass, or provision of habitats for plants and animals (Degerman et al. 2004; Trimble 2004; Dal Ferro et al. 2019). The vegetation in BZs can consist of grass for fodder production, or be natural vegetation with herbs, weeds, bushes and trees. The soil in these areas is often fertile since repeated floods have left behind fine-grained river sediments, and the land can therefore be valuable for the production of biomass for food, fodder, fuel, energy or materials. “The green shift” is a common expression used to describe the change from a world economy based on fossil fuel to a bioeconomy, and it is expected that the need for biomass will then increase (Hertel et al. 2012; O’Brien et al. 2017; Eyvindson et al. 2018). This, in turn, can increase the value of fertile land, and lead to an intensification of both agriculture and forestry (see Marttila et al., this issue, for a more comprehensive discussion on predicted land use changes following the transition to bioeconomy). BZs may then become increasingly important to reduce the pressure on water quality, but they may also be threatened, since this fertile land is valuable to farmers and land owners.

The effectiveness of BZs in retaining nutrients and soil particles has been explored by many authors (e.g. Dillaha et al. 1989; Liu et al. 2008; Hoffmann et al. 2009; Zhang et al. 2010; Roberts et al. 2012; Stutter et al. 2019). Phosphorus (P) is traditionally the most studied nutrient in Norwegian freshwater, not least because of high erosion rates of P-rich soils (Holtan et al. 1988, Faafeng and Hessen 1993). In this paper, we will therefore focus on the retention capacity for P in BZs. This capacity depends on several factors including vegetation, soil type, slope, hydrological conditions, the width of the zone, etc., which in turn can cause significant variations in the P-retention capacity. In a study of 11 BZs in Finland, Norway, Sweden and Denmark, Uusi-Kämpä et al. (2000) found that TP loads from agricultural runoff decreased by 27–97%. Similarly, Schmitt et al. (1999) found that BZs in the US reduced concentrations of TP by 55–79% and dissolved P (DP) by 19–43%. Former studies in Norway (Syversen 2002) estimated a retention of TP of 76–89% in 5–10 m wide grass-covered BZs with a slope of about 10%. In a review by Poulsen and Rubæk (2005), one of the studies had a negative TP retention of − 36%, but huge variations were reported, with the highest retention rate of + 97%. As such, the retention capacity of BZs has been questioned, for example in terms of their ability to remove DP (Roberts et al. 2012), not least when vegetation is not removed from the zones (Hoffmann et al. 2009; Stutter et al. 2009), or due to freeze–thaw processes in cold climate areas (Kieta et al. 2018). Furthermore, BZs only retain the nutrients entering through overland flow, and more sophisticated methods, such as integrated BZs and bioreactors (Zak et al. 2018; Zak et al. 2019; Carstensen et al., this issue) are therefore needed to also reduce runoff from ditches and tile drains. A disadvantage is that these more sophisticated measures may occupy more land than the traditional BZs, and hence further reduce the land available for cultivation.

Doubts about the retention capacity of BZs can reduce farmers’ motivation to implement them, as well as the likelihood that policy makers and managers ensure appropriate subsidies. An example is the fate of the Danish Buffer Zone Act, which was implemented in 2011, stating that buffer zones should be established on agricultural lands. For this reason, it becomes essential to also assess the other benefits of maintaining vegetation on these fertile strips of land. Bank erosion is naturally occurring in most rivers but can be enhanced by the removal of vegetation (e.g. Trimble 2004). Bank erosion not only causes loss of land and danger to infrastructures, but also increases water pollution by adding soil particles and associated P (Skarbøvik 2016). Reduced bank erosion in BZs with trees has been found by e.g. Micheli et al. (2004) and Pollen et al. (2004). Trees can also increase bank erosion locally, for example when they fall into the river and cause increased stream velocity, but this is, on the other hand, important for the ecology, as the trees provide food for benthic invertebrates and shade and shelter for fish (Degerman et al. 2004; Lie and Sørensen 2013).

Farmers have economic interests in the BZs, since these areas can have the most fertile soil in many farms, especially in areas with more marginal conditions for agriculture. As an example, Norway has only 3.5% agricultural land, and the requirement of land owners to cultivate as much as possible of the land suitable for agriculture is stated in the Norwegian Land Act. Furthermore, farmers only receive subsidies for land area on which they produce food or fodder, which means that BZs left with natural vegetation represent a net loss for farmers.

In this paper, our objective has been firstly to explore how different types of BZs function in terms of retention of P and as protection against bank erosion, and secondly how farmers perceive different types of BZs in terms of practical and economic issues. Our focus has been on grassed BZs (GBZ) usually used for fodder production, and BZs with natural vegetation (trees, bushes and herbs; NBZ). The paper presents a combination of new and hitherto unpublished data and a synopsis of field studies, questionnaires and interviews carried out as part of studies in South-Eastern Norway and published in national reports (Skarbøvik and Blankenberg 2014; Skaalsveen et al. 2015; Skarbøvik 2016; Blankenberg et al. 2017; Skarbøvik et al. 2018; Blankenberg and Skarbøvik 2018).

## Materials and methods

**Fig. 1:**
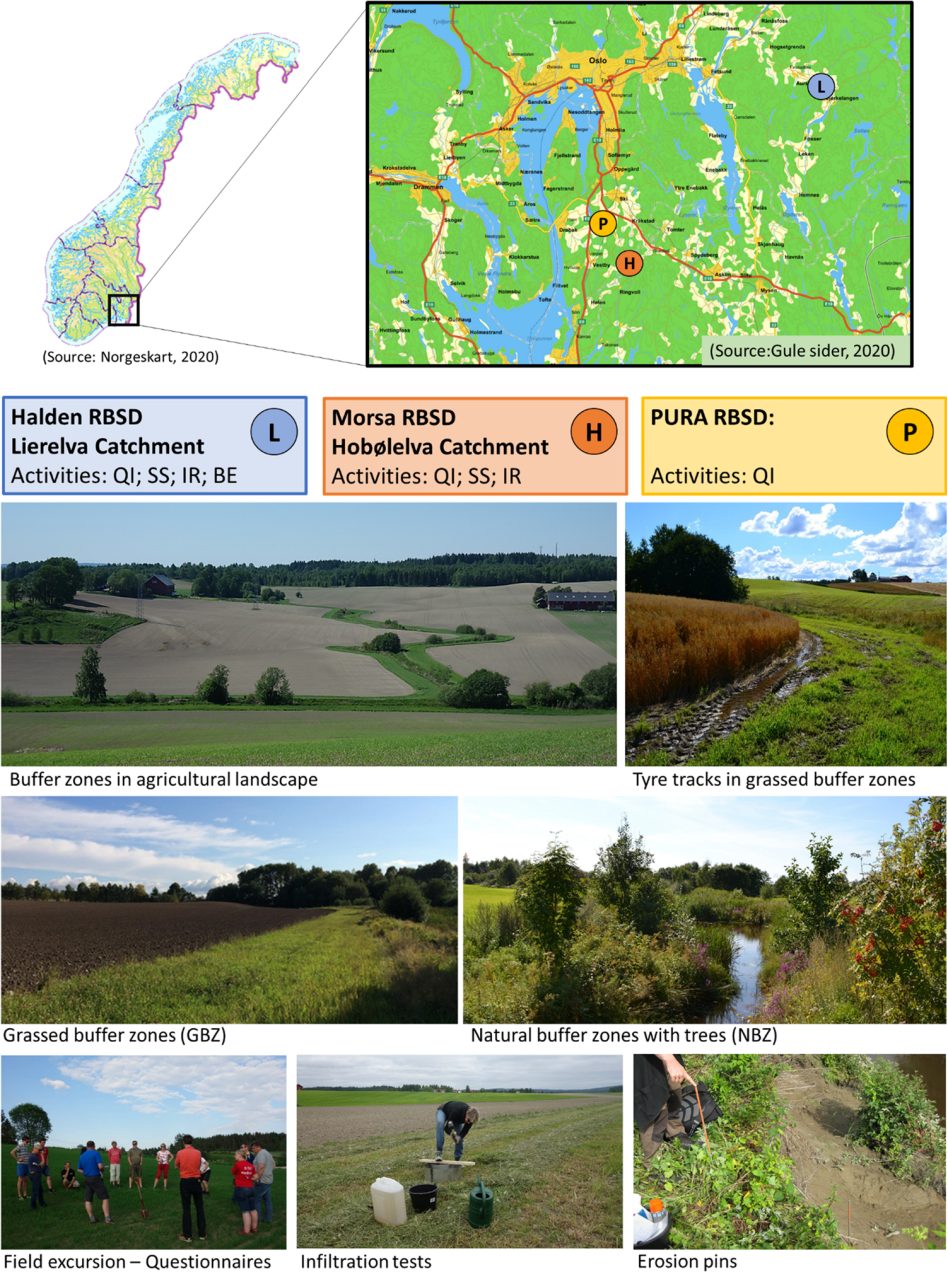
Location of the three case studies, the research activities performed in each of them, and photos from the sampling sites. *QI* questionnaires and interviews with farmers on BZs; *SS* soil sampling and analyses; *IR* infiltration rate tests; *BE* bank erosion. Photos: A–G. B. Blankenberg

**Fig. 2:**
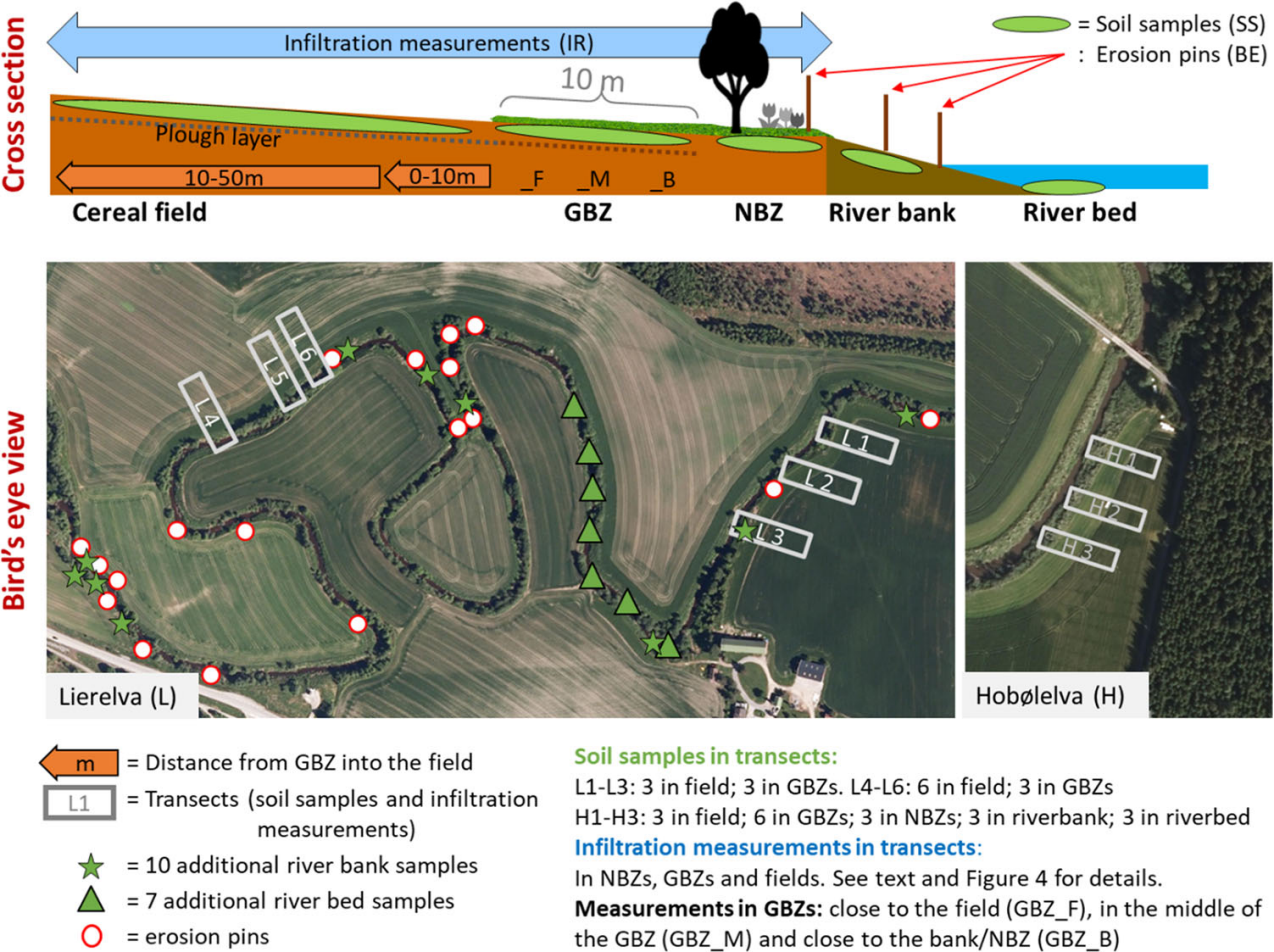
Cross-sectional sketch (top) of locations for soil sampling, infiltration tests and bank-erosion measurements. Bird’s eye view of transects L1–L6 in the Lierelva catchment (bottom left) and H1–H3 in the Hobølelva catchment (bottom right). Locations for bank-erosion measurements and additional soil samples from river bed and bank in the Lierelva catchment (bottom left)

Recent case studies in three river basin sub-districts (RBSD) in South-Eastern Norway have been used in this paper: the Halden, Morsa and PURA RBSDs. Figure 1 shows the location of the three study areas, the investigations performed in each of them and photos from the sampling sites. Interviews with farmers were performed in all three RBSDs, whereas field measurements were conducted in the Lierelva catchment in Halden RBSD and the Hobølelva catchment in Morsa RBSD, as illustrated in Fig. 2. All sites are located in relatively flat terrain with cereal production. According to the World Reference Base for Soil Resources (WBR), Hobølelva (H1–H3) is classified as a combination of Retic Stagnosol (Siltic) and Eutric Stagnosol (Siltic). Lierelva (L1–L3) is classified as Luvic Stagnosol (Siltic), and L4–L6 is classified as Retic Stagnosol (Siltic).

The GBZs were 8–10 metres wide, while the NBZs were narrower, about 2 metres. NBZs and GBZs often occurred together, with NBZs close to the river since legislation demands at least 2-metre-wide NBZs to be granted subsidies. The studied GBZs were established in the period 2008–2010. Tillage and sowing must take place between 1 March and 1 July and is usually only performed every fifth year. Hence, the GBZs have more constant vegetation cover than cereal fields that are harvested each autumn, and are either left in stubble over the winter or sown with winter wheat, depending on weather conditions. The grass in the GBZs is usually harvested twice a year. Commonly used seeds are *Phleum pratense, Festuca pratensis* and *Poa pratensis*. Until 2019, regulations allowed N fertilizer of up to 1 kg N/ha in the GBZs, but no P fertilizer application.

## Soil sampling for physical and chemical analyses

Altogether, 50 soil samples were collected from the cereal fields, GBZs, NBZs, riverbanks, and the river bed at two sites in the Lierelva catchment (transects L1–L3 and L4–L6) and one site in the Hobølelva catchment (transects H1–H3) during 2013 and 2014 (Fig. 2). The samples were collected from the plough layer in the fields and the GBZs, from 0 to 15 cm in the NBZs and river banks, and from 0 to 10 cm in the river bed. Each sample comprised 15–20 sub-samples mixed into a representative sample, except for river bed samples, where fewer sub-samples were collected due to practical constraints (the samples were collected from a canoe or from the banks with a collector attached to a long pole). Soil samples were sent to an accredited laboratory (NS-EN ISO 17025)^1^ directly after sampling, and analysed for total phosphorus (TP), phosphorus extracted with ammonium lactate (P-AL) and clay content. TP was analysed after extraction with 7 M nitric acid and determined with ICP-OES (ISO 11885 2016)^2^. The P-AL fraction is a commonly used parameter for plant-available (cereals, grass) phosphorus in Norwegian soils (Øgaard et al. 2012). P-AL is extracted with Ammonium Lactate (SS 028310) and determined with ICP-OES (ISO 11885 2016). Clay content is defined as the percentage of the clay particle size fraction. Particle size distribution was determined by sieving and sedimentation (ISO 11277 2015)^3^. Statistical analyses of the data on chemistry and clay content were performed using the program “R”.

### Infiltration rates

Infiltration rates were measured as steady-state infiltration with a double-ring infiltrometer (ASTM 2009). The tests were performed during 2013 and 2014 in three types of vegetation: cereal fields, GBZs and NBZs (Fig. 2). In the Lierelva sites, 29 infiltration tests were carried out during autumn (transects L1–L3) and 30 during spring (L1–L6). In the Hobølelva site, 12 infiltration tests were carried out during autumn, and three during spring (H1–H3). The variability of the data was analysed using the program “R”.

### Bank erosion

Erosion pins (e.g. Laubel et al. 1999) were inserted at 18 locations in river banks along both sides of a 3-km-long stretch of the Lierelva River. In general, one pin was inserted at the top of the bank (about 1 m from the brink), one halfway down the slope, and one close to the water level at low flow conditions (Fig. 2). A hose clamp was used to mark where the pin entered the soil, so that erosion could be measured as the increasing distance between the clamp and the bank sediments. Vegetation at the sites included NBZs with trees, GBZs and cereals (i.e. in sites with no NBZs or GBZs between field and river). The pins were inserted in June 2012 and were subsequently checked six times until September 2014. At each field visit, the river banks were photographed.

The erosion pin method functioned well in NBZs, but worked poorly in GBZs and in cereal fields, since the erosion there occurred as mass failure or slumping. Instead, the distance between the edge of the bank and the pin positioned in the BZ or the field was measured. The edges of the banks were quite sharply defined due to the slumping of the banks. When bank failure occurred, the new distance between the pin and the edge of the bank was measured and used, together with new measurements on-site, to calculate the total volume of the bank failure (Skarbøvik and Blankenberg 2014). The weight of the bank material was calculated by using a measured density of 1.5 kg/l.

### Farmers’ views of vegetation in buffer zones: survey

Two different questionnaires were developed: one on GBZs (distributed to approx. 145 farmers in two RBSDs; Halden and Morsa) and one on NBZs with trees (distributed to 29 farmers in two RBSDs; Morsa and PURA; see Fig. 1). The feedback on the questionnaires varied; we received 30 answers for GBZs and 18 answers for NBZs. Hence, 11 additional in-depth interviews were carried out on NBZs. In addition, we attended five different meetings in the three RBSDs where discussions with farmers on both GBZs and NBZs were facilitated by the RBSD managers. One of the surveys was performed in a municipality in the Morsa RBSD, where 10,000 trees had been planted along agricultural streams in the period 2001–2006. The questionnaires and interviews were carried out in the period 2014–2018. The topics of the questions are listed in Results section, in Table 1 (GBZs) and Fig. 6 (NBZs).

**Table 1:** Farmers’ written answers to the questionnaires on grassed buffer zones (GBZ) in two RBSDs

River basin sub-district (RBSD)	Halden	Morsa
Municipalities	3 (Aremark; Marker; Aurskog-Høland)	2 (Hobøl; Svinndal)
Number of filled-in questionnaires	10	20
Width of the GBZs (m)	6–12	6–12
	% Responded positively	
The GBZ is ploughed every (years)	3–8	b
Fertilizers are used in the GBZ	20	30
Pesticides are used in the GBZ	10	0
Grass is harvested in the GBZ	80	90
Grass is grazed by livestock in the GBZ	0	20
The GBZ is a financial gain	0	0
The GBZ is used as a transport route	30	15
Spreading of weeds from the GBZ is a problem	20a	b
GBZ reduces loss of soil and nutrients to water	60a	b
GBZ is perceived as an aesthetic element	100a	b
GBZ reduces bank erosion	100a	b
Infiltration is better in the GBZ than in the fields	100a	b

^a^Question was answered in writing in two of three municipalities

^b^No written answers

## Results

### P-retention capacity

#### Soil P chemistry

**Fig. 3:**
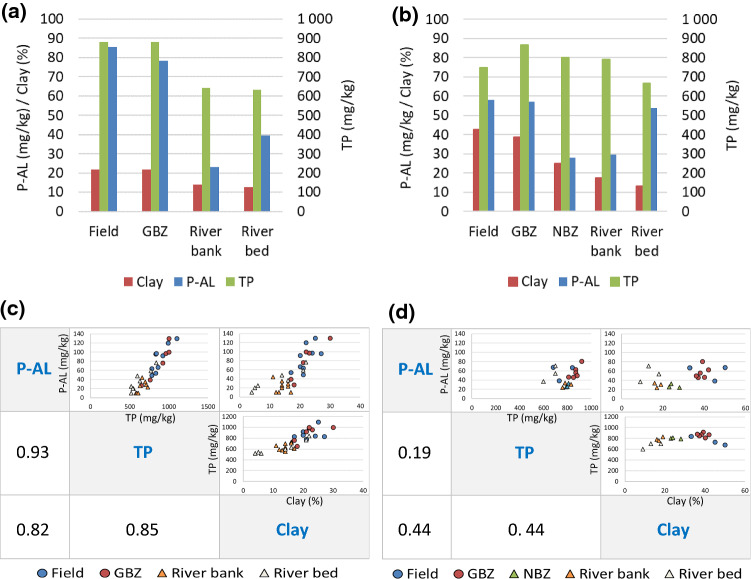
Average clay content (% dry weight) and phosphorus content (TP, P-AL; mg/kg) in soil samples from different sites in Lierelva (**a**) and Hobølelva (**b**); Pearson correlation between clay, TP and P-AL in all soil/sediment samples in Lierelva (**c**) and Hobølelva (**d**)

Average TP concentrations in the soils were just below 900 mg/kg in the cereal fields and GBZs in the Lierelva sites (Fig. 3a) and between 750 and 870 mg/kg in the fields, GBZ and NBZ in the Hobølelva site (Fig. 3b). The TP content of the fields was the same (Lierelva) or even lower (Hobølelva) than in the soil of the GBZs. The average P-AL levels in the fields and GBZs in the Lierelva sites were approx. 85 mg/kg and 78 mg/kg, respectively (Fig. 3a), while the average P-AL levels in the fields and GBZs in the Hobølelva site were approx. 58 mg/kg (Fig. 3b). The average P-AL levels in the NBZs (Hobølelva) and river banks (both catchments) were low (20–30 mg P-AL/kg). As shown in Fig. 3c, the Pearson correlation between clay content and TP in the Lierelva sites was good, while the correlation in the Hobølelva site was poorer (Fig. 3d).

#### Infiltration of water to the soil

**Fig. 4:**
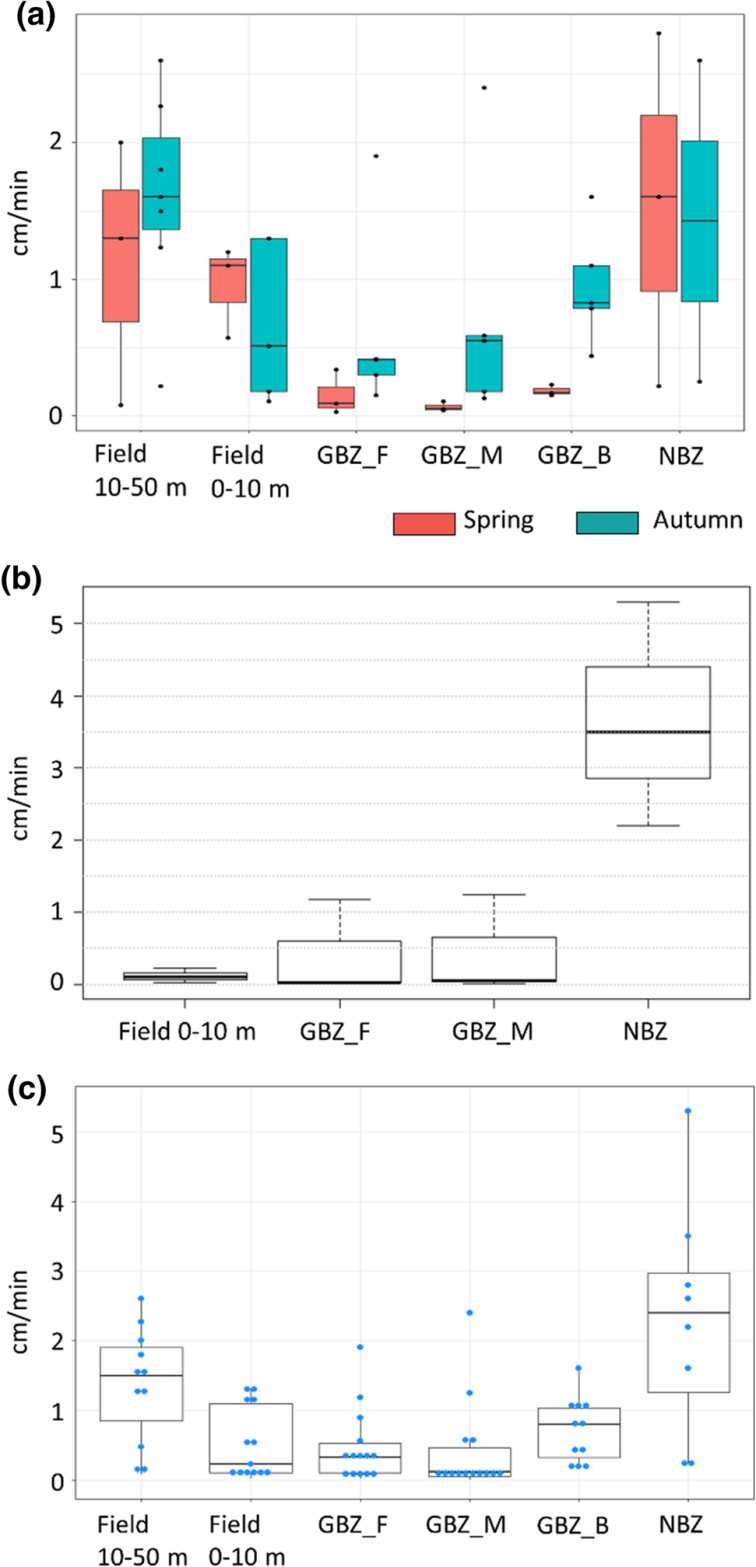
Infiltration rates (box plots/quantile plots) at two sites: Lierelva during spring and autumn (**a**), and Hobølelva during autumn (**b**). All samples are compiled in chart (**c**). Blue dots (**c**) are samples from both Lierelva and Hobølelva

Figure 4 shows the results of the infiltration tests in the Lierelva sites (spring and autumn) and in the Hobølelva site (autumn), as well as a compilation of all samples. Tests were also carried out in the Hobølelva site during spring but on dry soil with large cracks, and steady state was not reached. This demonstrated that water can occasionally infiltrate at high velocities due to soil cracks or piping along roots, with the risk of insufficient time for soil and plant processes to retain pollutants and nutrients.

The infiltration rates in the GBZs in the Lierelva sites were higher in autumn than in spring, probably due to better developed grass roots, but overall, the infiltration was poorer in the GBZs than in cereal fields and NBZs (Fig. 4a). In the Hobølelva site, the highest infiltration rates were found in the NBZs, followed by the GBZs, whereas the poorest infiltration rates were in the fields (Fig. 4b).

In general, the greatest variations in infiltration rates were found in the NBZs, while the lowest variations were found in GBZs (all samples taken into consideration; see Fig. 4c). On average for all three plots, the lowest infiltration rates were found in the middle of the GBZs (GBZ_M), followed by the cereal field closest to the GBZ (Field_ 0–10 m), the GBZs close to the field (GBZ_F) and GBZs close to the bank/NBZ (GBZ_B).

### Bank erosion

**Fig. 5:**
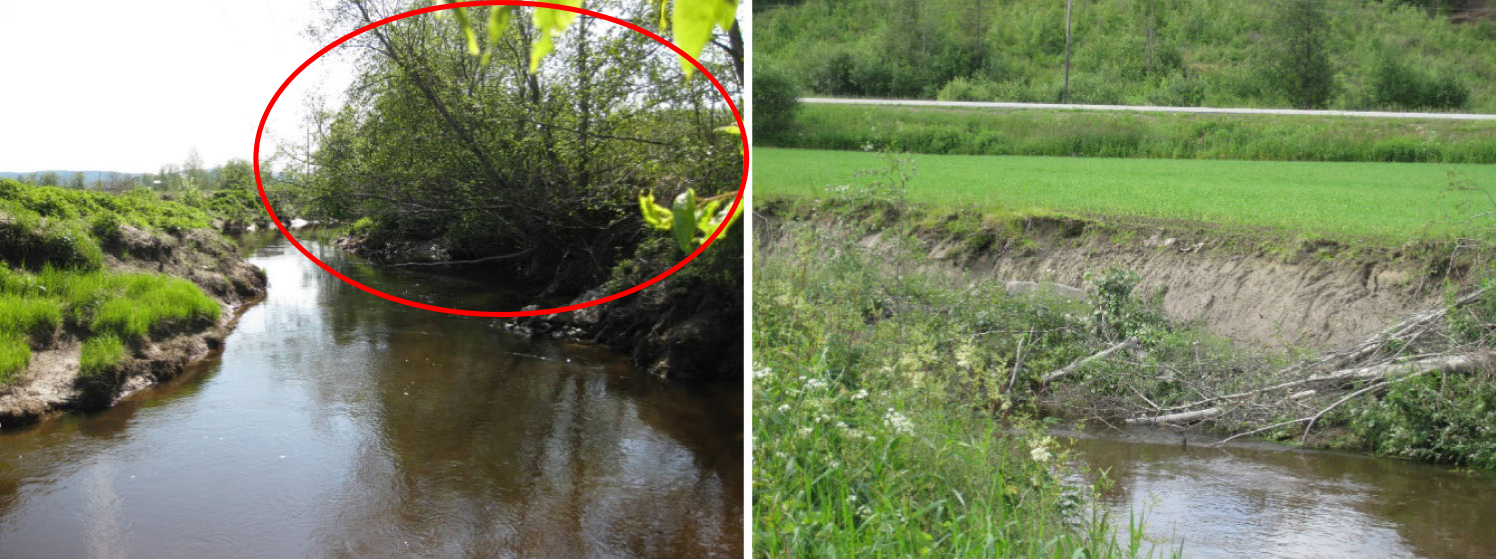
The importance of trees demonstrated in the Lierelva catchment. A row of trees (in the red circle, left-hand photo) were cut down along the river bank. A few months later, the logs had fallen into the stream, and a steep erosive slope had been formed (right-hand photo). Photos: E. Skarbøvik

In the Lierelva catchment, the average annual erosion per length of river bank (m) with trees (*n *= 7) was estimated to approx. 400 kg m^−1^, which was about half of that found in banks bordered by grass or cereal fields (approx. 775 kg m^−1^; *n *= 6). Five of the sites could not be used to calculate erosion rates because the pins had disappeared or been moved (probably due to farm activities along the river). Although the number of sites is too low to give a significant result, the differences in bank erosion between the vegetation types were considerable. Moreover, an episode in 2013 demonstrated the ability of trees to reduce bank erosion. An electricity company cut down a row of trees along 10 ms of river bank in April (Fig. 5), and 2 months later, a steep erosion slope had been formed at the site. In November the same year, about 20 m^3^ of soil had been lost into the river along this 10-m stretch, corresponding to approx. 3 tonnes m^−1^ of river bank. Based on the P levels of the river bank sediments (Fig. 3), the corresponding loss of TP from these 10 m was about 20 kg during these months.

### Farmers’ experiences and perspectives

**Fig. 6:**
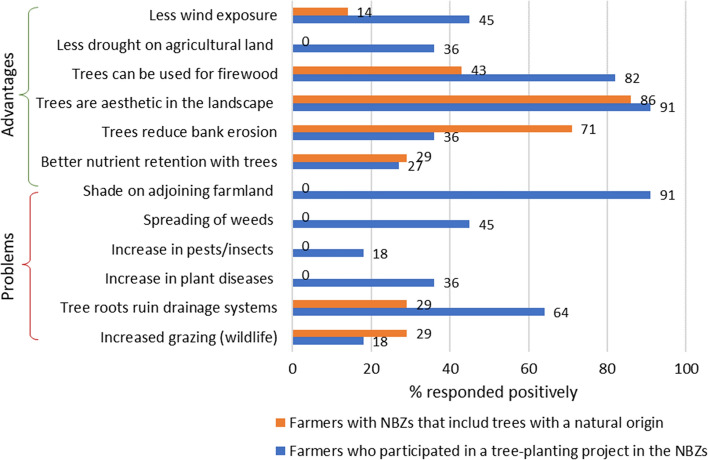
Farmers’ answers to questionnaires regarding NBZs (Morsa and PURA RBSD). In RBSD Morsa (*n *= 7), farmers had taken part in a project involving planting trees in the NBZs (blue rows); in RBSD PURA (*n *= 11), the trees in the BZs had a natural origin (orange rows)

Table 1 shows the farmers’ written assessments of the GBZs, whereas Fig. 6 shows written feedback of their views on NBZs. Feedback given orally during in-depth telephone interviews, in discussions during meetings or walks in the fields, are not included in Fig. 6 or Table 1, but were methodically noted down and are included in the discussion below.

For the GBZs, the general feedback was that most farmers perceive them as a necessary nuisance, probably good for the environment but less so for the farm economy (Table 1).

Less than a third of the farmers applied small amounts of N fertilizer (< 1 kg/ha) for improvement of the grass root system and soil structure, and hence increase the P retention through infiltration and uptake by plants. Most of the respondents stated that the grass was harvested, but very few had livestock that used the zone for grazing.

The most surprising finding from the questionnaires and interviews about trees in the NBZs was the difference in opinion among farmers who had planted trees and those who had not. There were many reasons for the negative attitude towards trees, including that the tree roots would ruin the drainage systems. Farmers were also concerned that trees would enhance plant diseases, pests and insects, augment the spreading of weeds, and increase the shade on adjoining fields. However, the farmers who had planted trees claimed that almost none of these perceived problems had been experienced. When questioned directly, all farmers who had planted trees said that they would have done it again, and some had already planned to plant more. Plant species included Betulaceae (*Betula pubescens, Betula pendula, Alnus glutinosa, Alnus incana);* Rosaceae *(Sorbus aucuparia, Prunus avium);* and Salicaceae (*Salix cinerea, Salix pentandra, Salix triandra, Salix alba, Salix phylicifolia, Salix myrsinifolia, Salix aurita).*

As for the advantages, most farmers felt that trees were aesthetic elements in the landscape, even if they might reduce the view. Most farmers also believed that tree roots could increase the infiltration capacity of the soil. Farmers who had planted trees claimed that the trees protected against bank erosion, whereas farmers who had not planted trees felt that local bank erosion increased when trees fell into the rivers. The latter farmers suggested that a combination of GBZs and mechanical enforcements (e.g. stones) would be a better form of bank-erosion protection.

## Discussion

The GBZs and the cereal fields studied in this investigation had comparable levels of plant-available P, measured as P-AL. The high TP levels in NBZs (Fig. 3b) can be linked to the high proportion of the P-rich mineral apatite in these soils. The variations in TP may be linked to the clay content in the samples. P-AL is of interest since water soluble P has been shown to increase with increasing P-AL concentration in the soil (Øgaard 1995; Krogstad and Løvstad 2002), indicating a link between soil P-AL and algae-available P in water. High P-AL values in soil can represent a significant source of pollution for water bodies, and 50–70 mg P-AL/kg is considered to be the optimal level for a combination of plant cultivation and water quality (Kristoffersen and Øgaard 2019). The P-AL levels in the fields and GBZs in the Hobølelva site were within this optimal level (Fig. 3b), while the P-AL levels in the fields and GBZs in the Lierelva sites were just above the optimal level (Fig. 3a). The comparable P-AL levels in fields and GBZs may have several explanations: (i) the relatively low P-AL of cereal fields may mask the retention effect of GBZs, meaning that the uptake of P in both cereals and grass has functioned well as a mitigation measure; (ii) the GBZs have received P from two sources (runoff from fields and deposition from floods), and this, combined with no/limited fertilizer application to the GBZs, has given the same P level as in the fertilized cereal fields; and/or (iii) the GBZs have been established recently and the effect is not yet visible. An answer can only be found by implementing new investigations of GBZs vs. NBZs, including in areas with higher P-AL levels, and over a longer period.

The water infiltration rates were on average poorer in the GBZs than in the fields. The reason is not fully known but tyre tracks were often observed in the buffer zones (Fig. 1) and driving on the GBZs was reported by farmers to be a better option than driving on bare and saturated fields with the subsequent risk of soil compaction and reduced cereal yields. Moreover, the harvested grass is often transported along the GBZs. The lower infiltration rates in fields close to the GBZs, as compared to fields 10–50 m from the GBZ, can also be linked to soil compaction, since this land is where the farmers turn the farm machinery (Obour et al. 2018).

Despite these results on soil chemistry and infiltration, GBZs may still play an important role in improving water quality, as the farmers do not spread fertilizers or manure close to the streams, and the ground is almost continuously protected against erosion by dense grass cover, except when the zones are ploughed and re-established. Previous studies have also shown that GBZs have significantly higher P retention in steeper terrain with higher soil P levels than were found in our cases (Syversen 2002).

NBZs had lower levels of P-AL, higher infiltration rates and lower banks erosion rates than both GBZs and cereal fields. The higher variability of infiltration rates in these zones may be explained with the natural variability, including soil cracks and plant roots of different sizes where the water can flow. Their role in enhancing biodiversity has been demonstrated by many authors (Gregory et al. 1991; Pusey and Arthington 2003), and since climate predictions for this part of Norway include higher precipitation and more extreme rainfall episodes (Hanssen-Bauer et al. 2015), their ability to retain nutrients and reduce erosion should be valuable. However, NBZs are not subsidized in Norway. Piégay et al. (2005) noted that several countries are changing their regulations so that afforestation along rivers is encouraged, but in countries where suitable land for agriculture is scarce, such regulations may still be far away. This calls for creativity, and the use of the BZs for berry bushes and fruit trees is being discussed, albeit when we launched this idea to farmers in the more informal interviews, most of them felt that this option was unlikely due to difficulties with harvesting.

In this part of Norway, the farms mainly produce cereals, and they have traditionally hardly had any livestock. Harvesting of the grass is important to prevent nutrient leakage from wilting plants during winter and early spring (Bechmann et al. 2005). In Morsa RBSD, a company had been established for grass production used for horse fodder, which rented out machinery for grass harvesting and managed sales of the fodder for its shareholders. However, the company soon experienced that GBZs are prone to floods, and the grass was often not usable for fodder due to fine-grained flood-sediments clinging to the grass (Sven Solberg, pers. comm.). Hence, the main message conveyed by the farmers was that GBZs were considered a financial burden. Despite this, most farmers preferred GBZs to NBZs, with a clear exception of the farmers who had planted trees along streams. The latter group were almost surprisingly enthusiastic about the trees. This may point to a certain prejudice against trees that is not entirely founded in practical experience, and suggests that dedicated information to farmers might be useful. A narrow row of trees or bushes (e.g. 3–4 metres wide) along water bodies may be better for both the environment and food production than 8–10 metre-wide GBZs often unfit for fodder production. However, more research is needed to conclude on this. NBZs with trees will also prevent transport of farm machinery along the rivers, and thereby reduce both bank erosion and the spreading of fertilizers close to water bodies. Most of the interviewed farmers preferred mechanical enforcements, but the added effects of trees on e.g. nutrient retention and biodiversity would in such case be lost. Pollen et al. (2004) noted a growing concern among managers of the environmental effects of streambank stabilization through mechanical measures such as steel and stone.

The assumed changes in land use due to increased need for biomass, combined with the predicted rise in precipitation and more frequent extreme weather episodes in this part of Europe, will increase the need for well-designed measures, both to prevent runoff from agricultural areas (Deelstra et al. 2011) and reduce bank erosion. In addition, the biodiversity of freshwaters should be considered, as trees along rivers and lakes provide food, shade and shelter. In the future, more integrated studies of BZs are therefore needed that consider the retention of nutrients; the prevention of bank erosion; the production of food, fodder and biomass; biodiversity on-site and downstream; as well as other ecosystem services such as recreation (Kenwick et al. 2009; Vidon et al. 2019). Table 2 gives an overview of such considerations, partly adjusted to the findings of this paper. More holistic considerations of this kind should form the basis for determining the subsidies granted to different types of BZs.

**Table 2:**
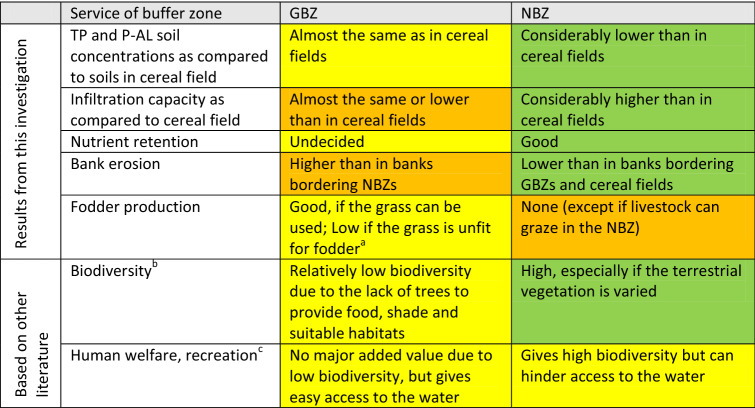
An evaluation of the benefits of the two types of BZs studied in this investigation, representing an example of how the authors feel that BZs should be assessed to ensure that their usefulness is explored in a holistic way

Colour codes: *Green* good effect; *Yellow* no effect; *Orange* negative effect

^a^According to the questionnaires and interviews with farmers, grass in buffer zones is often unfit for fodder since flood water can deposit fine-grained sediments on the stems; ^b^Gregory et al. (1991), Pusey and Arthington (2003);^c^Kenwick et al. (2009)

## Conclusions

The main findings of this work include the following:

- Plant-available P concentrations were comparable in the soils of the GBZs and the cereal fields, but lower in the NBZs. The GBZs had on average lower infiltration rates than both the cereal fields and the NBZs, possibly due to soil compaction from farm machinery. Hence, the ability of the GBZs to retain P in this type of land, with low slope and recommended P-AL-levels, is undecided.
- Bank erosion was higher in GBZs and cereal fields than in NBZs with trees.
- Despite subsidies, many farmers view GBZs as a financial loss since the grass was often unfit for fodder.
- In general, farmers who had been enrolled in a tree-planting scheme were more positive to having trees along the streams, whereas farmers who had not were more negative to their use. This points to the need for better information to farmers on this issue.

We propose that the usefulness and therefore also the subsidies for different types of BZs should be based on more holistic considerations, including nature (biodiversity, water quality), economy (production of food, fodder, biomass), as well as human wellbeing (recreation, health).

## Footnotes

1 https://www.eurofins.no/om-oss/kvalitet/akkreditering/.

2 https://www.iso.org/standard/36250.html.

3 https://www.iso.org/standard/54151.html.
